# Stability and flexibility of the gut microbiota of wild Tibetan macaques

**DOI:** 10.1093/ismeco/ycaf184

**Published:** 2025-11-26

**Authors:** Yangkai Ru, Wenbo Li, Paul A Garber, Yang Teng, Ming Li, Xiaochen Wang, Huijuan Pan

**Affiliations:** School of Ecology and Nature Conservation, Beijing Forestry University, Beijing 100083, China; State Key Laboratory of Animal Biodiversity Conservation and Integrated Pest Management, Institute of Zoology, Chinese Academy of Sciences, Beijing 100101, China; School of Resources and Environmental Engineering, Anhui University, Hefei, Anhui 230601, China; International Collaborative Research Center for Huangshan Biodiversity and Tibetan Macaque Behavioral Ecology, Anhui University, Hefei, Anhui 230601, China; Department of Anthropology and Program in Ecology, Evolution, and Conservation Biology, University of Illinois, Urbana-Champaign, IL 61801, United States; International Centre of Biodiversity and Primate Conservation, Dali University, Dali, Yunnan 671000, China; State Key Laboratory of Animal Biodiversity Conservation and Integrated Pest Management, Institute of Zoology, Chinese Academy of Sciences, Beijing 100101, China; State Key Laboratory of Animal Biodiversity Conservation and Integrated Pest Management, Institute of Zoology, Chinese Academy of Sciences, Beijing 100101, China; State Key Laboratory of Animal Biodiversity Conservation and Integrated Pest Management, Institute of Zoology, Chinese Academy of Sciences, Beijing 100101, China; School of Ecology and Nature Conservation, Beijing Forestry University, Beijing 100083, China

**Keywords:** *Macaca thibetana*, 16S rRNA gene, DNA metabarcoding, dietary, ecological adaptation

## Abstract

The gut microbiota of wild animals is characterized by both stability and adaptive shifts in composition and prevalence in response to variation in food availability, nutrient intake, host physiology, temperature, and rainfall. Here, over a 12-month period, we investigated seasonal interactions between diet, weather, and gut microbiota in a wild group of Tibetan macaques in Huangshan by recording feeding behavior, monitoring weather, and analyzing 209 fecal samples using plant DNA metabarcoding (*trn*L region) and 16S rRNA gene sequencing. Based on the field observations and plant DNA metabarcoding, results revealed marked seasonal shifts in plant types and species consumed by Tibetan macaques. Despite dietary variability, only two enterotypes were presented throughout the year and gut microbiota composition exhibited lower dissimilarity within and across seasons compared to diet, except in autumn when low dietary diversity correlated with reduced microbial diversity. In addition, we also found that the enrichment of seasonal indicator bacterial genera and functions was related to the temperature or the nutrients of the food consumed by Tibetan macaques during that season. This study highlights the microbiota’s resilience and metabolic plasticity in buffering seasonal dietary shifts, underscoring its role in maintaining host energy homeostasis under fluctuating resource availability.

## Graphical Abstract

**Graphical Abstract:**
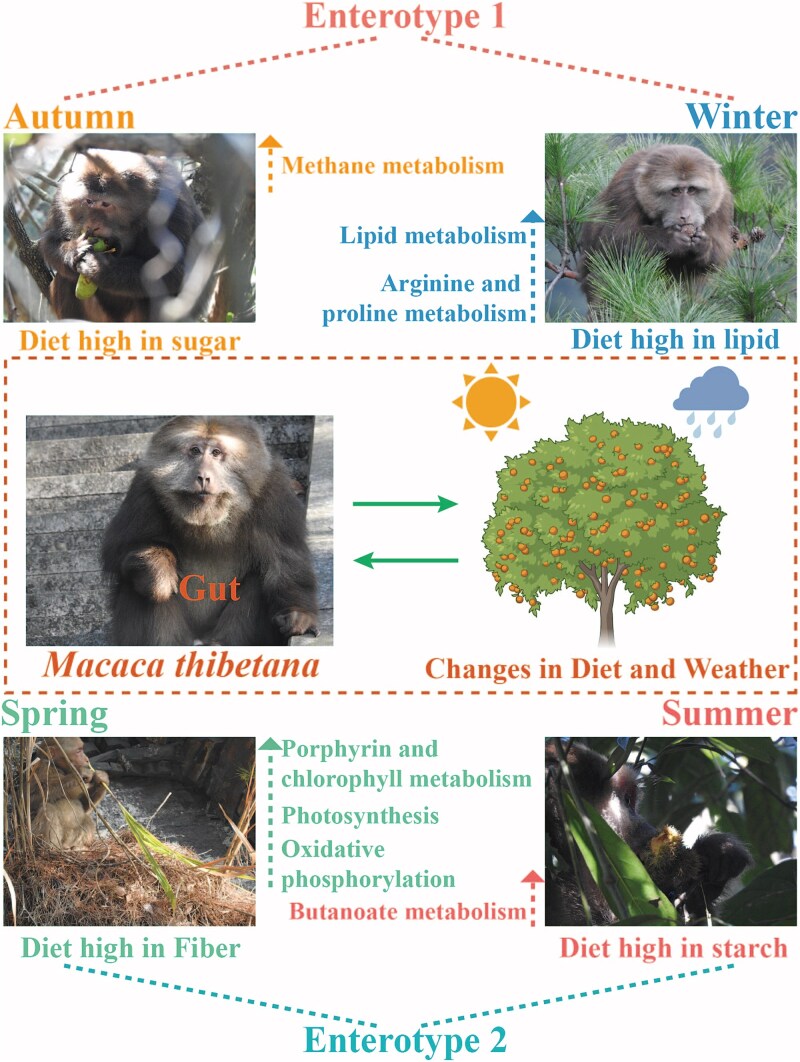

## Introduction

The gastrointestinal tract of mammals represents a complex and changing microbial ecosystem composed of both prokaryotes and eukaryotes, that impacts host health, nutrition, reproduction, growth, and development [1–3]. The number of genes encoded by the gut microbiota significantly exceeds the number of genes in the cells of their host’s tissues, which further expands the capability of the microbiota to metabolize substances such as dietary fiber, complex carbohydrates, and xenobiotics, and modulate the host immune system [4]. In this regard, the gut microbiota can be thought of as a virtual organ of the host, producing and regulating essential compounds like short-chain fatty acids, vitamins, and neurotransmitters such as serotonin [5, 6]. The ability of the gut microbiota to vary in composition, taxonomic richness, diversity, and function in response to host diet, physiology, and ecology offers critical insight into the adaptive strategies employed by wild mammals in adjusting to seasonal changes in the nutritional and toxic content of foods consumed and the energetic demands of their environment [2, 7].

A primary function of the gut microbiota is to more effectively digest and absorb nutrients by fermenting dietary fiber and starch, participating in protein metabolism, and influencing lipid absorption and metabolism [1, 2, 8, 9]. In some species, such as François’ langurs (*Trachypithecus francoisi*) and koalas (*Phascolarctos cinereus*) [10, 11], the gut microbiota is highly conservative and may show limited taxonomic variation across different periods of the year. This is mainly due to their specialized dietary patterns, which formed a stable community system in the intestine [10, 11]. In other species, however, the gut microbiota is more dynamic and exhibits significant temporal shifts in composition, diversity, and or richness in response to changes in host diet and local environmental conditions. For example, the family Prevotellaceae was enriched in Skywalker gibbons (*Hoolock tianxing*) when fruits dominated their diet, while Lachnospiraceae and Oscillospiraceae increased with higher leaf consumption [12]. In addition, the gut microbiota of wild plateau yaks *(Bos grunniens*) and pikas (*Ochotona curzoiae*) was found to be affected by changes in ambient temperature [13, 14]. During the winter, cold exposure selects for gut enterotypes in these species that enhance nitrogen/energy utilization, thermoregulation, and energy metabolism [13, 14]. Taken together, these studies highlight a set of dietary, nutritional, and climatic factors that can affect the stability and variability of the mammalian gut microbiota.

In order to better understand the relationship among host gut microbiota, diet, and weather, we studied the behavior and microbial ecology of a wild nonprovisioned and fully habituated group of 23 Tibetan macaques (*M. thibetana*) living at Mt. Huangshan, China. Mt. Huangshan has a subtropical monsoon climate, and exhibits marked seasonal variation in temperature, rainfall, and plant phenology [15]. Tibetan macaques (*Macaca*, Cercopithecidae, and Primates) are endemic to China, and have been listed as a Class II protected species since 1988. They are categorized as Near Threatened by the International Union for the Conservation of Nature and have a declining population (IUCN Red List, 2024). Although previous study has compared the gut microbiota of Tibetan macaques in Huangshan between winter and spring seasons, this study of Tibetan macaques largely focused on provisioned groups and quantitative data on their natural diet are not collected [16]. This research gap potentially weakens our understanding of the fine-scale relationship between Tibetan macaque gut microbiota and their natural environment.

Thus, in our 12-month study, we monitored the local weather conditions and quantified the proportion of different food species and types in the Tibetan macaque diet by behavioral observations. In addition, we used plant DNA metabarcoding and 16S rRNA gene sequencing to identify the taxonomic composition of plants consumed and gut microbiota in Tibetan macaques. Compared with field observations, plant DNA metabarcoding cannot provide information about plant types such as fruits, leaves, or flowers, but it offers more comprehensive, individualized dietary data and facilitates correlation analysis with gut microbiota data. Our study aims to characterize the seasonal dynamics of gut microbiota in wild Tibetan macaques, and determine how their gut microbiota responds to their diet and weather conditions.

## Materials and methods

### Study site and animals

Our research was conducted in the Niejiashan area, which is located at the junction of Mt. Huangshan and the Tianhu Nature Reserve, in Anhui Province, China (Fig. S1). The study site includes large and intact broad-leaved evergreen forests and mixed deciduous and broad-leaved evergreen forests [17, 18]. A previous study, based on camera trapping data, identified at least 18 mammal species, including two primate species: *M. thibetana* and *M. mulatta*, which inhabit the study area [19].

During the study period (October 2022–September 2023), we collected temperature, rainfall, and humidity from an automatic weather station (QS-3000) at 1-h intervals. The weather station was located 300 meters from the border of the study group’s home range. Daily temperature and humidity were averaged from hourly recordings. Monthly mean temperature and humidity were calculated from daily averages, and monthly rainfall was calculated as the sum of hourly rainfall that occurred in that month. Following Li *et al.* [15], four seasons were defined: autumn (October–November 2022), winter (December 2022–February 2023), spring (March–May 2023), and summer (June–September 2023). In our study area, the warmest, wettest, and most humid period of the year was summer (mean temperature was 26.1 ± 1.4°C, rainfall was 948 mm, and humidity was 81.9 ± 1.4%). The coldest (4.6 ± 1.3°C) and least humid (71.3 ± 5.2%) period was winter. The driest season was autumn (169 mm) (Fig. S2).

Our study subjects were a group of wild Tibetan macaques (TianhuII Group). The group was initially surveyed in 2018 and has been continuously tracked and observed since then. To facilitate the location of the macaque group, we equipped a GPS collar on one adult male and one adult female group member. This study group principally inhabited forests at altitudes ranging from 200 to 600 m. During the study period, group size fluctuated from 19 to 27 due to immigrations, emigrations, deaths, and births. The number of adult males ranged from four to six, adult females from seven to eight, subadults from one to three, juveniles from six to nine, and infants from 0 to two.

### Feeding behavior observation

We located macaques based on their previous day’s sleeping site or by tracking the signal transmitted by their GPS collars in the early morning. Given that the monkeys ranged across dense forests and steep mountains passages, it was sometimes difficult to continuously follow the study group. When the group entered the sleeping site at dusk or was lost for a period of >30 min, daily observations ended. We used an instantaneous scan sampling method at 10-min intervals to record the behavioral activities of as many individuals as possible [20]. When individuals were feeding, we recorded the food species and food type consumed. Throughout the study period, we obtained 8680 individual feeding records from 2805 scans over 62 days. On average we collected more than 6 h of behavioral observations per day (Table S1).

### Fecal sample collection

During the process of tracking the monkeys, fresh fecal samples free of soil and litter contamination were collected within 10 min of defecation. Samples were placed into 50-ml sterile tubes with 95% ethanol [21] and stored at −20°C until the end of the day’s tracking. Then temporarily-preserved samples were transported to the laboratory in dry ice and subsequently stored in a −80°C freezer before nucleic acid extraction. During the study period, 209 fecal samples (17.4 ± 9.1 per month) were collected (Table S1). We used 0.2 g feces for DNA extraction with E.Z.N.A.®stool DNA Kit (Omega Bio-tek, Norcross, GA, USA) following the manufacturer’s instructions. The DNA samples (*n =* 209) were used for both identification of plant species consumed and microbiota analyses.

### Diet DNA metabarcoding and reference plant DNA libraries

To assess the plant components in the macaque diet, the P6 loop of the chloroplast *trn*L (UAA) region was used to amplify the extracted DNA, with primers *trn*L-g forward (5′-GGGCAATCCTGAGCCAA-3′) and *trn*L-h reverse (5′-CCATTGAGTCTCTGCACCTATC-3′) [22]. PCR reactions were performed in triplicate in a 20 μl mixture containing 2 μl of template DNA (10–50 ng/μl), 10 μl of 2 × Taq Master Mix, 0.5 μl of each primer (10 μm), and 7 μl of ddH_2_O. Thermocycling followed a program of initial denaturing at 95°C for 3 min, followed by 30 cycles at 95°C for 20 s, 50°C for 15 s, and 72°C for 30 s, with a 5-min final extension at 72°C. The 5′ end of each primer was tagged by an 8-nt multiplex identification tag, allowing for sample differentiation. The PCR products were carried out on an Illumina MiSeq platform using 150 paired-end reads (Shanghai BIOZERON Co., Ltd.).

Raw sequence data demultiplexing, quality filtering, and preliminary identifications were conducted by QIIME 2. Reads were quality-trimmed using Trimmomatic with a 20-bp sliding window. We eliminated sequences with an average score <25, and discarded any resulting sequences that contained <150 bp. Paired-end (PE) reads were assembled based on overlaps >10 bp, with a maximum mismatch rate of 0.2 within the overlapping regions. Primer sequences were removed, and sequences shorter than 100 bp were filtered out using the plug-in “cutadapt” in QIIME2. Chimeric sequences and redundant sequences were removed using DADA2. Finally, the resulting sequences were clustered to generate amplicon sequence variants (ASVs).

Based on the Huangshan plant directory [23], the sequences of the *trn*L region of plants were retrieved from NCBI database, and a local DNA reference database with a total of 1483 plant species was established using BLAST+ 2.15.0. More details about the construction of reference database are shown in supplementary methods.

The *trn*L barcode sequencing results were compared for similarity against the established plant databases, and the taxon annotation principles are shown in supplementary methods.

### 16S rRNA gene Illumina sequencing

The V3-V4 regions of the DNA genes were amplified by using the universal primer 341F (5′-CCTAYGGGRBGCASCAG-3′) and 806R (5′-GGACTACHVGGGTWTCTAAT-3′) [13]. The PCR cycles started with a 5-min denaturation at 95°C, followed by 30 cycles at 95°C for 30 s, 58°C for 30 s, 72°C for 45 s, and a final extension at 72°C for 10 min. 20 μl reactions of 4 μl of 5 × FastPfu Buffer, 0.8 μl of each primer (5 μm), 2.5 μl of dNTPs (2.5 mM/L), 4 μl of FastPfu Polymerase, and 10 ng of template DNA were mixed. The amplicons were sequenced on Illumina HiSeq and generated 2 × 250 bp paired-end reads.

We used the same method as detailed above to perform quality control, denoising, merging, and dereplication on the raw data of the bacterial 16S rRNA gene, obtaining clean reads. Each unique sequence generated was referred to as an ASV at a 100% similarity threshold. Based on Silva _132 databases, we trained a classifier against the bacterial V3-V4 region of the 16S rRNA gene, and used this classifier to generate a classification map of our data [24].

### Statistical analysis

Due to inconsistencies in data collection across months and daily variation in the starting and ending times of data collection, following Zhang *et al.* [25] we divided the number of individuals consuming a food type in each scan by the total number of individuals recorded as feeding for that scan. Then, we averaged the data for six scans per hour and generated an hourly percentage of time spent feeding on each food type. Next, we took the average of the hourly data for the number of hours we followed the monkeys that day to represent the daily percentage of time spent feeding on each food type. Finally, the average daily data was used to create monthly, seasonal, and yearly profiles.

We examined the variation in relative abundance across groups, and the Kruskal–Wallis test (multiple groups) or Wilcoxon test (two-sample comparisons) was used to assess significant differences within the profiles in standard R commands (*stats* package; version 4.3.1). A Mantel test was used to evaluate the correlation between the matrix of bacterial genera abundance and vectors of multiple environmental factors in the *vegan* package. We computed Bray–Curtis dissimilarity and applied a nonmetric multidimensional scaling (NMDS) analysis for both the diversity of plant taxa consumed and microbiota, using ASV data within the *vegan* package [26]. Permutational multivariate analysis of variance (PERMANOVA) was used to investigate the differences in Bray–Curtis dissimilarity among seasons. We employed Procrustes analysis using *vegan* package, to compare the relative positions of points in two multivariate datasets to assess the relationship between the taxonomic diversity of plant species consumed and microbiota composition [27]. To examine the alpha diversity of the plant taxa composition of the diet and microbiota, richness, Chao1 and Shannon indices were calculated and plotted in R. Within each season, the correlation between the alpha diversity of plant taxa consumed and microbiota in each sample was examined through linear regression analysis.

The clustering of enterotypes in Tibetan macaque datasets was identified using Jensen–Shannon divergence (JSD) and partitioning around medoid (PAM) clustering in R [28]. The robustness of the clusters was evaluated using the Calinski–Harabasz (CH) index and the silhouette width was calculated using the *clustersim* and *cluster* packages, respectively, in R [29]. We performed the between-class analysis (BCA) with *ade4* in R to identify which taxa contributed to the enterotype [30].

We used Phylogenetic Investigation of Communities by Reconstruction of Unobserved States (version 2; PICRUSt 2), based on the KEGG database, to predict the functional profile of the microbiota [31]. Linear discriminant analysis effect size (LEfSe) (https://www.omicstudio.cn/tool) was used to detect the genus and functional guilds (KEGG pathways level 1 involves metabolic pathways) with significant differences among seasons and between enterotypes. Bacterial genera with LDA scores over 3.5 for four seasons and *P* < .05 were retained. Similarly, the functional guilds with LDA scores over 2 for enterotypes and 2.5 for the four seasons were retained. Furthermore, to determine which plant taxa and microbial taxa were most responsible for seasonal differences in diet and microbiota variations, we employed indicator species analysis using *indicspecies* package in R [32]. We applied the multipatt function with 9999 permutations to identify species of each season and used the r.g. function to calculate the correlation between binary vectors. SankeyMATIC (http://sankeymatic.com/) was used to visualize the indicator species across seasons.

## Results

### Seasonal dietary types and species consumed examined by field observations

**Figure 1:**
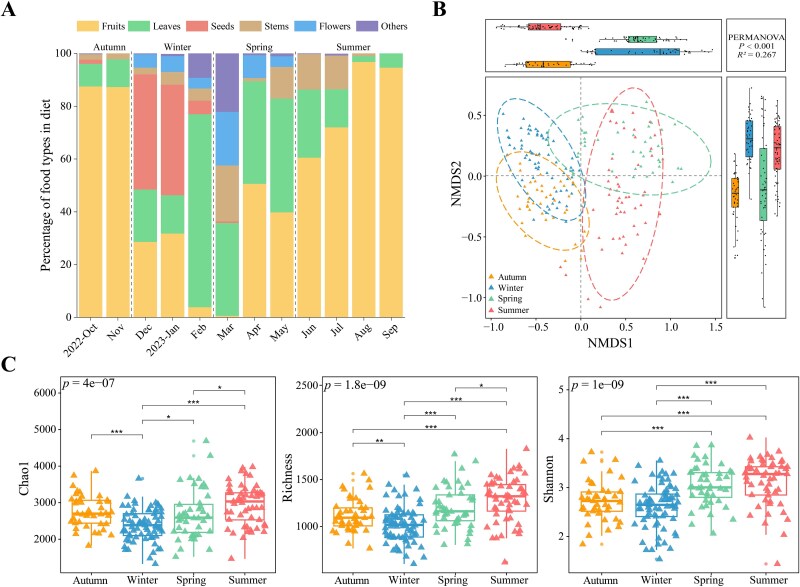
Seasonal variation in plant-based diet consumed by wild Tibetan macaques. (A) Monthly variation in the percentage of food types consumed by Tibetan macaques based on observation from October 2022 to September 2023. (B) Variation in beta diversity of the plant species composition of the diet across seasons based on Bray–Curtis dissimilarity. The ellipses represent a 95% confidence interval for each group. Significance was set at the 0.05 level. The left and the upper boxplots are drawn by season based on the MDS1 and MDS2 values, respectively. The left and the upper boxplots are drawn by season based on the MDS1 and MDS2 values, respectively. (C) Variation in alpha diversity, including Chao1 index, richness, and Shannon index, of the plant species composition of the diet across seasons. The boxplot distributions are tested using a nonparametric Kruskal–Wallis test and a Wilcoxon test with FDR (false discovery rate) and corrected *P*-values. Center values indicate the median and error bars. ^*^P < .05, ^**^P < .01, ^***^P < .001.

During the 12-month study period, the Tibetan macaque diet consisted of fruits (54.5%), leaves (24.3%), seeds (7.7%), stems (6.5%), flowers (4.0%), buds (2.5%), bark (0.2%), and roots (0.1%). Invertebrates/vertebrates such as insects, bird eggs and pupae in dry beehives accounted for 0.2% of feeding time. Fruits, seeds, and buds consumed by the Tibetan macaques varied significantly across seasons (fruits: *F* = 9.571, *N* = 12, *P* = .005; seeds: *F* = 5.687, *N* = 12, *P* = .022; buds: *F* = 4.567, *N* = 12, *P* = .038) (Fig. 1A). Fruits accounted for over 87.4% of feeding time in autumn and 81.0% in summer. In winter, leaves (30.2%), seeds (30.2%), and fruits (21.4%) dominated the diet. In spring, leaves (39.1%), fruits (30.3%), stems (11.5%), and flowers (11.0%) were the most common food types consumed (Fig. 1A).

Based on the field observations, the seasonal dietary diversity index was lowest in autumn (Shannon diversity index =1.43) and highest in spring (3.19). Dietary diversity in winter (2.43) and summer (2.93) were intermediate. During autumn, 68.8% of Tibetan macaque feeding time focused on the consumption of a single species of fruit, *Stauntonia brachyanthera*. In winter, the top three plant species accounted for 56.4% of the feeding time, with the seeds of *Pinus massoniana* accounting for 36.1% of the diet. In contrast, no single plant species accounted for more than 20% of feeding time during spring and summer (Table S2).

### Plant composition and diversity across seasons examined by DNA metabarcoding

Based on metabarcoding, we obtained a total of 14 508 219 clean reads (69 417 ± 8877 sequences per sample) that clustered to 42 506 ASVs. These were assigned to 74 plant families, 180 genera, and 130 species. We found that the plant species composition of the Tibetan macaque diet differed across seasons (Fig. 1B; Fig. S4). The Shannon diversity index of plant species composition was significantly lower in autumn and winter than in spring and summer (Fig. 1C). The top three plant families in autumn (Fabaceae 48.9%, Fagaceae 17.0%, and Myrtaceae 13.4%) and winter (Myrtaceae 37.7%, Fabaceae 28.4%, and Smilacaceae 8.8%), accounted for 79.3% and 74.9% of the ASVs, respectively. In contrast, in spring (Myrtaceae 18.3%, Rosaceae 15.0%, and Smilacaceae 14.4%) and summer (Fagaceae 12.5%, Fabaceae 11.9%, and Rosaceae 10.2%), the top three plant families accounted for less than 50% of ASVs (Fig. S3). We found that the top indicator plant family each season, based on their relative abundance and frequency of occurrence in the fecal samples, were Fabaceae in autumn, Myrtaceae in winter, and Moraceae in both spring (10.2%) and summer (9.0%) (Fig. S5).

### Gut microbiota composition and diversity across seasons

**Figure 2:**
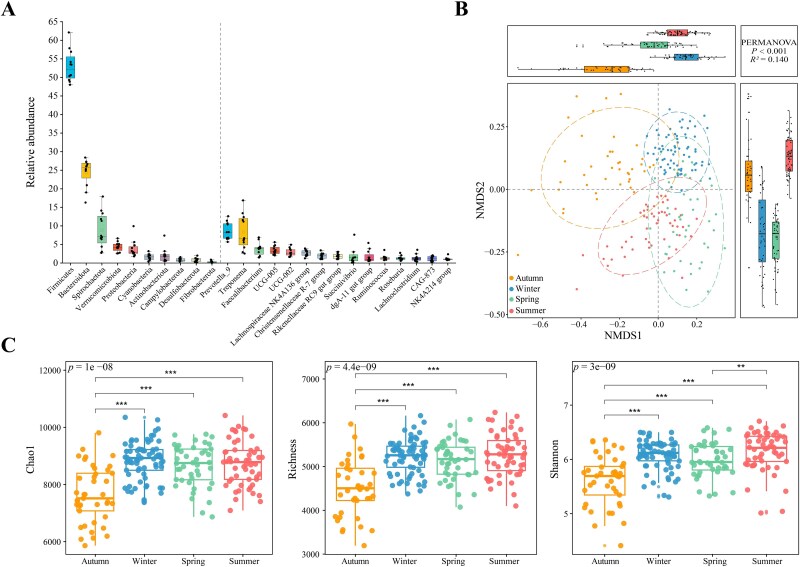
Fecal microbiota composition and seasonal alpha and beta diversities of Tibetan macaques. (A) Relative abundance (left of dotted line) of the 10 most abundant bacterial phyla (relative abundance ≥0.84%) and (right of dotted line) of the 15 most abundant families (relative abundance ≥1.42%) in the Tibetan macaque feces. (B) The variation in beta diversity of the gut microbiota across seasons was based on Bray–Curtis dissimilarity. The ellipses represent a 95% confidence interval for each group. Significance was set at the 0.05 level. The left and the upper boxplots are drawn by season based on the MDS1 and MDS2 values, respectively. (C) The variation in alpha diversity, including Chao1 index, richness, and Shannon index, of the gut microbiota across seasons. The boxplot distributions are tested using the nonparametric Kruskal–Wallis test and Wilcoxon test with FDR (false discovery rate) with corrected *P*-values. Center values indicate the median and error bars. ^*^P < .05, ^**^P < .01, ^***^P < .001.

The resulting amplifications yielded 15 580 507 clean reads (74 548 ± 5455 sequences per sample) and 39 705 ASVs. These ASVs were divided into 35 phyla, 75 classes, 175 orders, 289 families, and 576 genera. Over a 12-month period, the top five most abundant bacterial phyla in the feces of Tibetan macaques were Firmicutes (53.1 ± 4.0%), Bacteroidota (24.8 ± 4.1%), Spirochaetota (8.7 ± 4.7%), Verrucomicrobiota (4.3 ± 1.3%), and Proteobacteria (3.6 ± 2.1%). At the genus level, their microbiota was dominated by *Prevotella_9* (8.7 ± 2.2%), *Treponema* (8.3 ± 4.4%), *Faecalibacterium* (3.8 ± 1.6%), *UCG-005* (3.4 ± 1.1%), and *UCG-002* (2.9 ± 1.0%) (Fig. 2A).

Our results indicated that 17 bacterial phyla exhibited significant differences in abundance across seasons (Table S3). The remaining 18 phyla were present in low abundance (<0.01%) during all seasons. We found that indicator bacterial phyla per season were Spirochaetota and Proteobacteria in autumn and Bacteroidota in winter, spring, and summer (Fig. S5). In addition, we detected significant seasonal variation in bacterial beta diversity, suggesting that Tibetan macaque gut microbiota composition varied seasonally (Fig. 2B; Fig. S4). Bacterial alpha diversity was less variable, and three alpha diversity indices in autumn were all significantly lower than those in other seasons (Fig. 2C).

### The factors affecting gut microbiota composition and diversity

**Figure 3:**
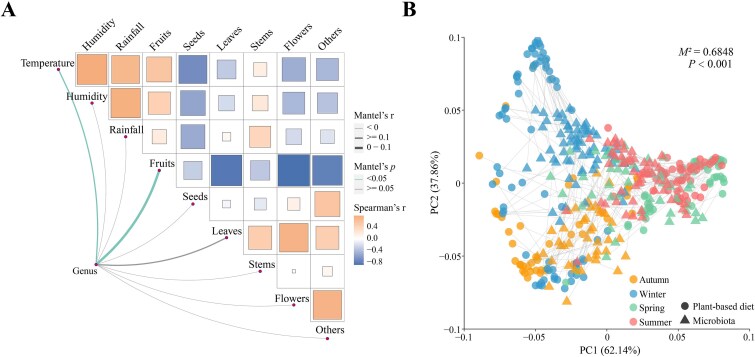
Diet-microbiota lineages of Tibetan macques. (A) Correlation analysis among bacterial genera, food types consumed, and weather conditions. The values are shown in Table S4. (B) The plant-based diet and microbiota composition were determined using Bray–Curtis dissimilarity, which are colored by seasons. Procrustes rotates the results of separate principal coordinates of plant species composition (circle symbols) and gut microbiota composition (triangle symbols). Significant dissimilarities occurred between plant-based diet and microbiota ordinations based on *P*-values.

We examined the degree to which the food type consumed, temperature, humidity, and/or rainfall affected the taxonomic composition of the gut microbiota of Tibetan macaque. Our findings revealed that mean temperature and time spent feeding on fruits were positively correlated with the bacterial genera abundance matrices (Fig. 3A, Fig. S6, Table S4). We next examined the degree to which these factors affected gut microbiota diversity. The results showed that whereas increased time spent feeding on fruits had a significant negative correlation with microbiome diversity (Chao1: *R^2^* = −0.204, *P* = .003; richness: *R^2^* = −0.177, *P* = .010; Shannon: *R^2^* = −0.144, *P* = .038) (Table S5), increased time spent feeding on leaves had a significant positive effect on gut microbiota diversity (Chao1: *R^2^* = 0.206, *P* = .003; richness: *R^2^* = 0.195, *P* = .005; Shannon: *R^2^* = 0.158, *P* = .022) (Table S5).

In order to better understand the relationship between seasonal variations in dietary and microbiota diversity (based on ASVs), we calculated the mean Bray–Curtis dissimilarities, both within and between seasons. For diet, the within-season dissimilarity ranged from 0.570 in autumn to 0.789 in summer (Table 1). Although fruits dominated the diet in both autumn and summer, the higher value in summer indicated an increase in the number of plant species consumed.

**Table 1:** Bray–Curtis dissimilarities in diet and gut microbiota of Tibetan macaques within and among seasons. Pairwise distance matrices denote the mean weighted Bray–Curtis dissimilarities of diets (lower triangle) and gut microbiota (upper triangle) across all samples during each season. Values represent the weighted means of Bray–Curtis dissimilarities. These values range from 0 to 1. The closer the value is to 0, the smaller the difference; the closer to 1, the greater the difference.

Season	Autumn	Winter	Spring	Summer	Within-seasons
Autumn		0.655	0.671	0.663	0.610
Winter	0.682		0.631	0.626	0.552
Spring	0.896	0.895		0.627	0.611
Summer	0.899	0.907	0.811		0.587
Within-seasons	0.570	0.600	0.747	0.789	

In terms of the gut microbiota, within-season dissimilarity ranged from 0.552 in winter to 0.610 in summer, indicating a more limited range of variability (Table 1). A comparison between seasons also indicated greater variability in dietary ASVs (0.225) compared to microbiota ASVs (0.045). The dissimilarity index for the plant species composition of the diet ranged from 0.682 between autumn and winter to 0.907 between winter and summer (Table 1). The seasonal dissimilarity in the gut microbiota ranged from 0.626 between winter and summer to 0.671 between autumn and winter (Table 1). Overall, we found a pattern of greater within- and between-season similarity in the gut microbiota compared to the plant species composition of the Tibetan macaque diet.

We next examined evidence of dissimilarity in the plant species consumed by Tibetan macaques and gut microbiota. Our results indicate that dissimilarity in plant species composition of the diet during the summer (within-season) and between spring and summer did not differ significantly (*P* = .140). In contrast, within season variability in diet and microbiota composition was always significantly lower than the between-season variation (*P* < .001) (Table 1, Table S6).

A Procrustes analysis indicated a significant correlation between seasonal variation in gut microbiota composition and seasonal variation in the taxonomic diversity of the Tibetan macaque diet (Fig. 3B). We found that within and between all seasons, the composition of the gut microbiota was always found to be less variable than the plant taxonomic composition of the diet (*P* < .001) (Table 1, Table S7). The only exception was autumn, when variability in the gut microbiota was significantly higher than the variation in the plant composition of the diet (*P* = .002) (Table S7). Finally, we examined the degree to which within-season alpha diversity of both the gut microbiota ASVs and diet ASVs co-varied across seasons. The results indicate that although the diversity indices of the gut microbiota and diet were positively correlated in each season, it was only during summer that the Shannon diversity indices of the ASVs showed a significant relationship (*R^2^* = 0.098, *P* = .022) (Fig. S7).

### Indicator bacterial genera and predicted function of microbiota in each season

**Figure 4:**
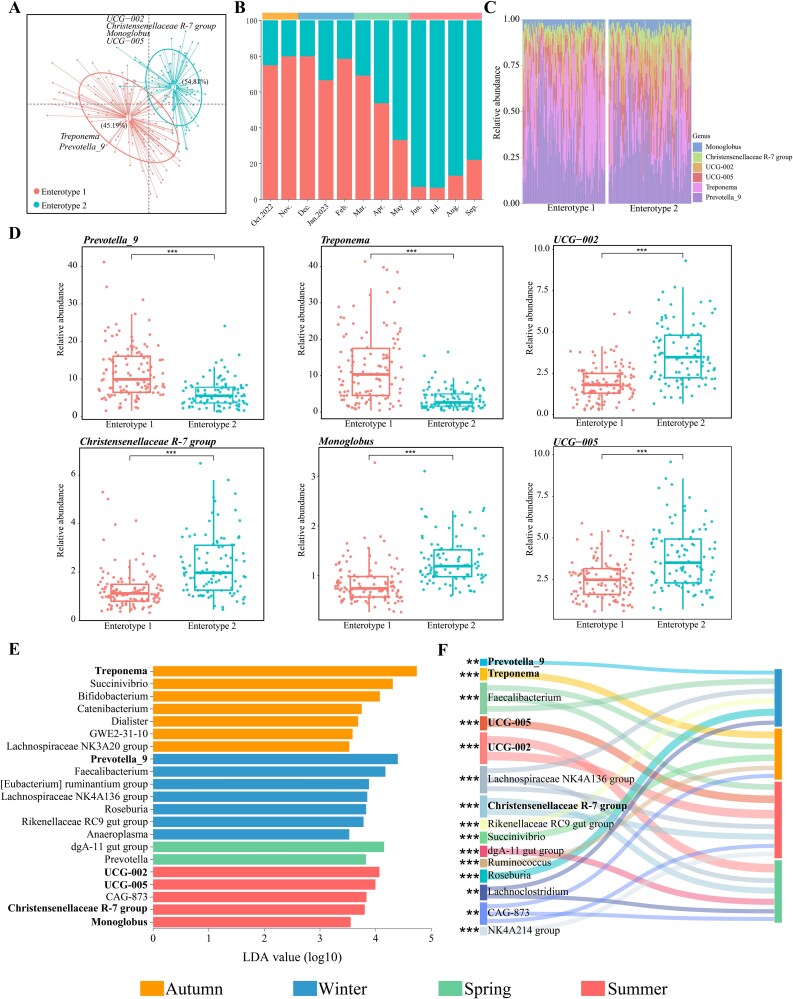
Clustering of gut microbial taxa into two enterotypes and indicator species of different periods. (A) Visualizations of enterotypes, as identified by PAM clustering, with genera corresponding to each enterotype identified by their relative abundance. The dataset represents the percentage of samples belonging to a particular enterotype out of all samples. (B) Proportion of samples for each enterotype each month. (C) Relative abundance of bacterial taxa characteristics of each enterotype. (D) The relative abundances correspond to the main contributors at the gut microbiota genus level. Boxplot center values represent the median plus error bars. These six genera were chosen based on their average contribution to overall Jensen–Shannon divergence. All boxplot distributions were examined using Wilcoxon tests with FDR (false discovery rate) and corrected *P*-values. (E) Indicators of bacterial genera among different periods identified by LEfSe analysis (LDA > 3.5, *P* < .05). (F) Indicator genera that are related to each season by *indicspecies* and visualized using Sankey plots. Lines represent associations between indicator genera and seasons, which are colored by genus level. Line width is scaled to reflect indicator value (higher indicator value indicates that the genus was more strongly associated with that season). Indicator values are shown in Fig. S11. The statistical *p* values indicates whether the genus was associated with that period. ^*^P < .05, ^**^P < .01, ^***^P < .001.

Given that different enterotypes may serve different functions in breaking down particular nutrients, we explored evidence of seasonal differences in microbiota composition. We identified two enterotypes in wild Tibetan macaques. Enterotype 1 was characterized by the representative bacterial genera: *Treponema* and *Prevotella_9*, which accounted for 45.2% of all samples. Enterotype 2: *UCG-002*, *Christensenellaceae R-7 group*, *Monoglobus*, and *UCG-005*, accounted for 54.8% (Fig. 4A, Fig. S8). These representative genera, and alpha-diversity metrics, including richness (*P* = .022) and the Shannon diversity index (*P* = .003), all showed significant variation between enterotypes (Fig. 4C and D, Fig. S9, Fig. S10). Moreover, the distribution of different enterotypes varied across seasons. Enterotype 1 was primarily present in autumn, winter, and early spring (ranging from 69.8% to 80.0%), whereas enterotype 2 was primarily present in late spring and summer (ranging from 66.7% to 93.3%) (Fig. 4B). This seasonal partitioning of enterotypes was consistent with the bacterial Bray–Curtis dissimilarity patterns (Table 1).

We employed LEfSe analysis and *indicspecies* to identify indicator bacterial genera in each season. The results showed that *Treponema* was an indicator genus associated with enterotype 1 in autumn and *Prevotella_9* was an indicator genus associated with enterotype 1 during the winter. In contrast, each of the representative genera for enterotype 2 were indicator genera only during the summer (Figs. 4E, F, Fig. S11). The indicator genera in spring were the *dgA-11 gut group* and *Prevotella* (Fig. 4E).

**Figure 5:**
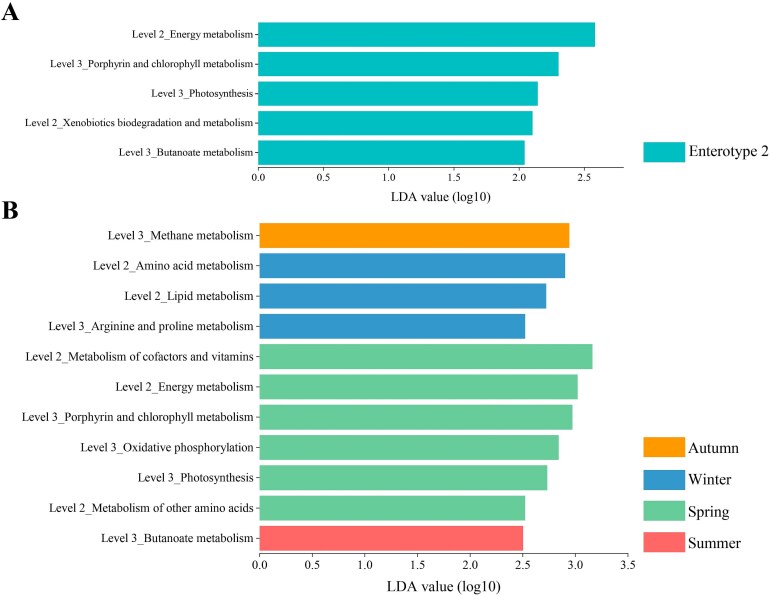
Metabolic pathway-enriched predicted functional guilds by enterotypes and seasons. (A) Between two enterotypes (LDA > 2, *P* < .05). (B) Among the four seasons (LDA > 2.5, *P* < .05). Level 2_KEGG-level-2 category; level 3_KEGG-level-3 category.

We next examined the functional guilds (KEGG, predicted pathways level 1 involves metabolism) that differed significantly between enterotypes. LEfSe analyses revealed that two level-2 metabolic functional pathways (energy metabolism and xenobiotics biodegradation and metabolism) and three level-3 pathways (porphyrin and chlorophyll metabolism, photosynthesis, and butanoate metabolism) were overrepresented in enterotype 2. In contrast, there were no enriched level 2 and level 3 metabolic functional pathways associated with enterotype 1 (Fig. 5A). Considering that the representative bacterial genus in enterotype 1 in autumn was *Treponema* and in winter was *Prevotella_9*, this may obscure gut microbial function related to weather and diet. This is due to the fact that such clustering prioritizes taxonomic abundance variation rather than functional divergence, which may be driven by environmental factors. Therefore, we focused on evidence of significant seasonal differences in KEGG predicted pathways. The level-2 metabolic functional pathways of amino acid metabolism and lipid metabolism were enriched in winter, whereas the metabolism of cofactors and vitamins, energy metabolism, and metabolism of other amino acids were enriched in spring. For level 3, three KEGG predicted pathways, including porphyrin and chlorophyll metabolism, oxidative phosphorylation, and photosynthesis, were overrepresented in spring. Finally, methane metabolism, arginine and proline metabolism, and butanoate metabolism were enriched in autumn, winter, and summer, respectively (Fig. 5B).

## Discussion

Resources consumed by wild animals vary in time, space, quantity, and nutrient and toxin content [33]. To meet the nutritional and energetic demands, foragers can rely on the synergistic relationship with the gut microbiota to collaboratively break down and metabolize complex dietary components from plant and animal tissues [21, 34, 35]. Our study found that across seasons, the food types and plant taxa consumed by Tibetan macaques exhibited greater variation than did their microbial communities, which tended to remain relatively stable. We found that time spent feeding on fruits had a significant negative effect on gut microbial diversity, whereas time spent feeding on leaves was found to increased microbial diversity. In addition, we identified two distinct enterotypes in the Tibetan macaque gut microbiota, as well as indicator bacterial genera and KEGG predicted pathways in different seasons. Overall, the Tibetan macaque microbiota exhibited limited within and between season variability, however, there was evidence of seasonal community bacterial restructuring that resulted in critical changes in gut function.

Our results indicate that Tibetan macaque dietary diversity was lower in autumn and winter and higher in spring and summer. This is consistent with our field observations and as well as a 12-month study by Li *et al.* [15] of this same Tibetan macaque group conducted two years earlier. However, the Tibetan macaque bacterial dissimilarity within and between seasons was consistently lower than dietary dissimilarity, except in autumn, suggesting that the Tibetan macaque gut microbiota exhibited strong resilience in response to seasonal fluctuations in diet. This is consistent with a study of wild Assamese macaques (*M. assamensis*) which found that despite marked seasonal changes in diet (79.7% leaves in the dry season and 50.1% fruit in the rainy season), microbiota diversity remained relatively stable [36]. Microbial community resilience appears to be driven by metabolic plasticity, which allows a core set of bacteria to adapt to dietary changes by adjusting their enzyme activity and patterns of metabolite secretion, rather than relying on the actions of other bacterial strains [37]. For example, in lean individuals, carbohydrate degradation is primarily driven by the genus *Prevotella*, while in obese individuals, the same metabolic role is fulfilled by *Ruminococcus* through distinct glycoside hydrolases like cellulases and amylases [38]. In addition, a previous study indicated that in healthy adults, gut microbiota composition fully recovers within 72 h after reverting from a high-fat to a high-fiber diet, demonstrating microbial resilience through rapid functional taxon resurgence [39].

In contrast, under conditions in which ecological stresses surpass the microbiota’s capacity to resist, their repair mechanisms cannot counteract disruptions. This can result in the collapse of the community’s equilibrium resilience [40]. In the case of Tibetan macaques, we note that only during autumn, the alpha diversity of the gut microbiota was lowest and Bray–Curtis dissimilarity in the gut microbiota was higher than the plant species composition of the diet. This appears to be best explained by a marked reduction in dietary diversity. In the autumn, a more specialized diet possibly limited the range of fermentable substrates available to the gut microbiota and prioritizing the enrichment of a narrow cohort of high-efficiency taxa, while reducing the abundance of other commensal bacteria [41]. The pattern we found of low dietary diversity driving low microbiota diversity in Tibetan macaques is consistent with observations of several other primate species such as white-headed langurs (*T. leucocephalus*), geladas (*Theropithecus gelada*), and rhesus macaques (*M. mulatta*) [42–44].

Although the Tibetan macaque gut microbiota exhibited less variation compared with the diet, we found that The indicator genera underwent a shift between different seasons. Based on a literature review, we found evidence that the nutrient composition is a factor driving seasonal changes in microbiota composition and function.

In autumn, fruits of *S. brachyanthera* dominated the diet. The husk of this plant species contains 32.6% sugar and 20.1% pectin [45], and the ripe fruit pulp contains 78.7%–82.8% water and 14.3%–19.3% total sugar [46]. The significantly enriched methane metabolism we detected in the Tibetan macaque microbiota during the autumn appears to be the result of the sugar and water content of their diet. Short-chain carbohydrates, can create an osmotic effect in the small intestine, drawing water into the large intestine, where they are fermented by methanogenic archaea to produce hydrogen and methane, thereby increasing the host’s methane metabolism [47, 48]. *Treponema* was found to be the indicator bacterial genus in autumn. A previous study found that some species of *Treponema* break down plant polysaccharides such as pectin, arabinogalactan, starch, and inulin as fermentable substrates [49]. Additional evidence from studies of pigs and rumens indicate a positive relationship between *Treponema* and sugars, especially pectin [50, 51].

During the winter, low temperatures may pose significant thermoregulatory and energetic challenges to primate species [52]. In the case of Tibetan macaques, we found that during the coldest period of the year, pine seeds (*P. massoniana*) accounted for over 30% of feeding time. These pine seeds contained 55.5% lipids [53], and their gut microbiota at this time was characterized by increased functionality in lipid metabolism. Lipids contribute approximately twice the energy per gram as do carbohydrates and proteins [52, 54, 55]. In addition, we identified enriched arginine and proline metabolism during winter in the Tibetan macaques. Arginine and proline help hosts adapt to low temperatures by protecting cells by lowering their freezing point, maintaining osmotic balance, providing energy, stabilizing protein structures, regulating cellular signal transduction, and promoting ATP production under cold stress [56].

In spring, leaves and stems (50.6%), which contain a large proportion of fiber (structural carbohydrates), were the most common food types consumed by Tibetan macaques [57–59]. At this time, we found that the macaque gut microbiota was enriched oxidative phosphorylation. A recent study found that the diet deficient in dietary fiber can result in deficiency in functions related to oxidative phosphorylation [60]. Oxidative phosphorylation acts to nourish the hypoxic environment of the intestines, which is beneficial for the fermentation of dietary fiber by obligate anaerobes [60]. In addition, *Prevotella*, which was enriched during spring has been found to strongly correlate with the consumption of a high fiber intake [61]. *Prevotella* increased in mice after arabinoxylan (a polysaccharide in fiber) supplementation, and has a higher prevalence in humans consuming plant-rich, fiber-abundant diets [61].

During summer, similar to autumn, Tibetan macaques spent over 80% of their feeding time on fruits, but the indicator genera, functionality, and enterotypes of their gut microbiota showed distinct patterns compared to autumn. This is likely primarily due to the significant differences in nutrient intake between seasons [21, 62]. The fruits which dominated Tibetan macaque diet in summer are found to contain high total starch (38.6%–62.8%), with digestive-resistant starches accounting for ~70% of the total starches content [63–65]. Resistant starches, instead of being digested or absorbed in the small intestine, move directly into the large intestines, where they serve as a carbon source for microbiota, including the Oscillospiraceae that produce butyrate as part of the butyrate kinase-mediated pathway [66, 67]. A recent study found that the increased butyrate concentration in the gut of Liaoning cashmere goats (*Capra hircus*), led to the proliferation of *Oscillospiraceae UCG-005* and the *Christensenellaceae R-7 group* [68]. These findings are consistent with our findings of indicator genera (*Oscillospiraceae UCG-002*, *UCG-005,* and the *Christensenellaceae R-7 group*) and enriched functions (butanoate metabolism) in summer. Thus, a diet assessment conducted at a broad scale (e.g. lumping all fruit species into a single category), may not accurately predict variations in the gut microbiota, or that the percent time spent feeding may not be a strong measure of the amount of food intake or the amount of specific nutrients ingested.

## Conclusions

In conclusion, combining the results of plant DNA metabarcoding and field observations, we found that both within and across seasons, variation in the food types and plant species consumed by members of a wild group of Tibetan macaques was greater than variation in the composition of their gut microbiota. We found only two enterotypes were present throughout the year, and the alpha diversity of gut microbiota remained relatively stable during most seasons. We detected seasonal enriched indicator bacterial genera and predicted function, which were mainly related to the food consumed by Tibetan macaques and the temperature in each season., We acknowledge certain limitation in our study. Considering our large number of the fecal samples, we may have insufficient sequencing depth and the reliance on predictive functional profiling rather than precise functional genetic data. Additionally, the gut microbiota is also influenced by individual hosts, but we were unable to accurately identify the host of each fecal sample. Nevertheless, we believe that our study still demonstrates how wild Tibetan macaques leverage gut microbial plasticity in response to seasonal changes in the nutrient content of their diet, and offers new insights into the evolutionary synergy between host dietary ecology and microbiome-mediated adaptation in nonhuman primates [21, 69].

## Supplementary Material

Supplementary_Figures_and_Tables_ycaf184

Supplementary_Methods_ycaf184

## Data Availability

The local DNA reference database is available at Zenodo (https://zenodo.org/records/17309411). The sequencing data presented in the study are deposited in the National Genomics Data Center (NGDC, https://ngdc.cncb.ac.cn/gsa/), accession numbers CRA023648 and CRA023558. Source values underlying the figures can be found in supplementary data.
