# Supplementary material for: Stability and flexibility of the gut microbiota of wild Tibetan macaques
Source: ISME Commun. 2025 Nov 26;5(1):ycaf184. doi: 10.1093/ismeco/ycaf184 (PMC12648401; doi:10.1093/ismeco/ycaf184)
Supplement: Supplementary_Figures_and_Tables_ycaf184 [file supplementary_figures_and_tables_ycaf184.pdf]

Supplementary figures

Supplementary Fig. 1 The map indicates the sampling locations of the 209 fecal samples of Tibetan macaques (TianhuII Group) in the Niejiashan area, Huangshan City, Anhui Province, China. The different seasons are represented by different colored dots: autumn: yellow (n = 41), winter: blue (n = 71), spring: green (n = 44), summer: red (n = 53).

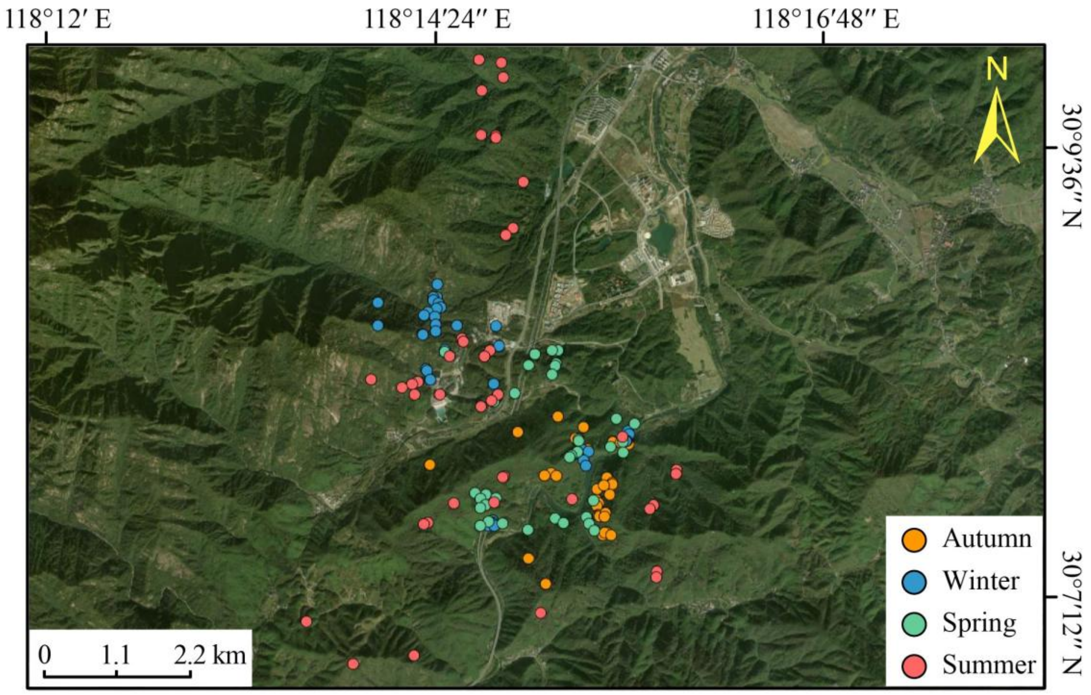

Supplementary Fig. 2 Weather conditions including mean monthly temperature, humidity, and rainfall for sampling region from October 2022 to September 2023.

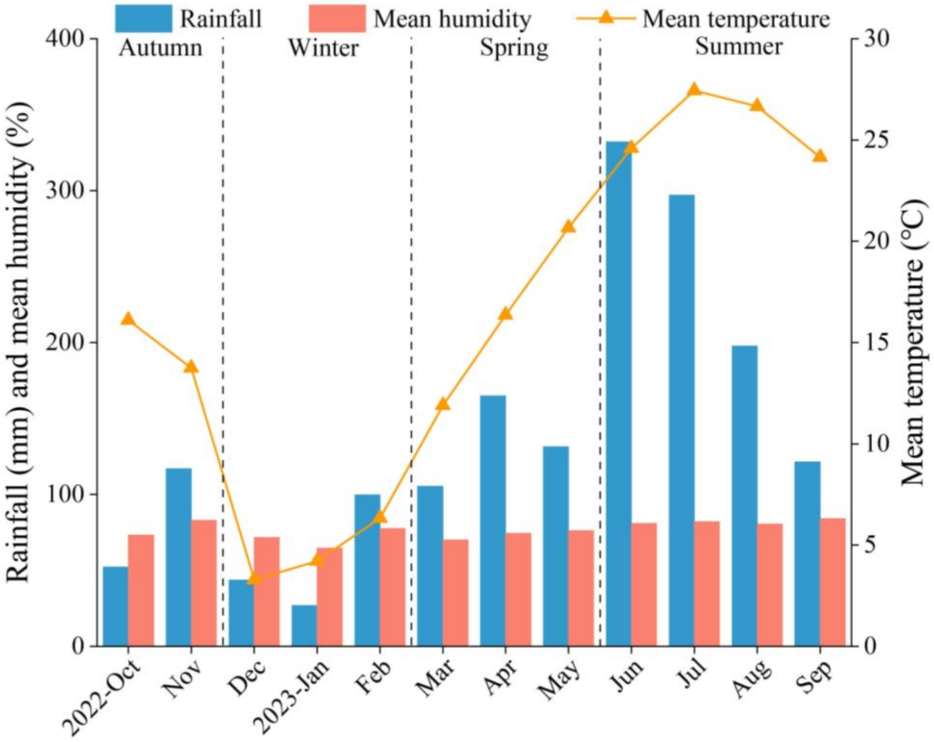

Supplementary Fig. 3 Monthly variation in the relative abundance of top 10 plant family-level taxa throughout the year examined by plant DNA metabarcoding.

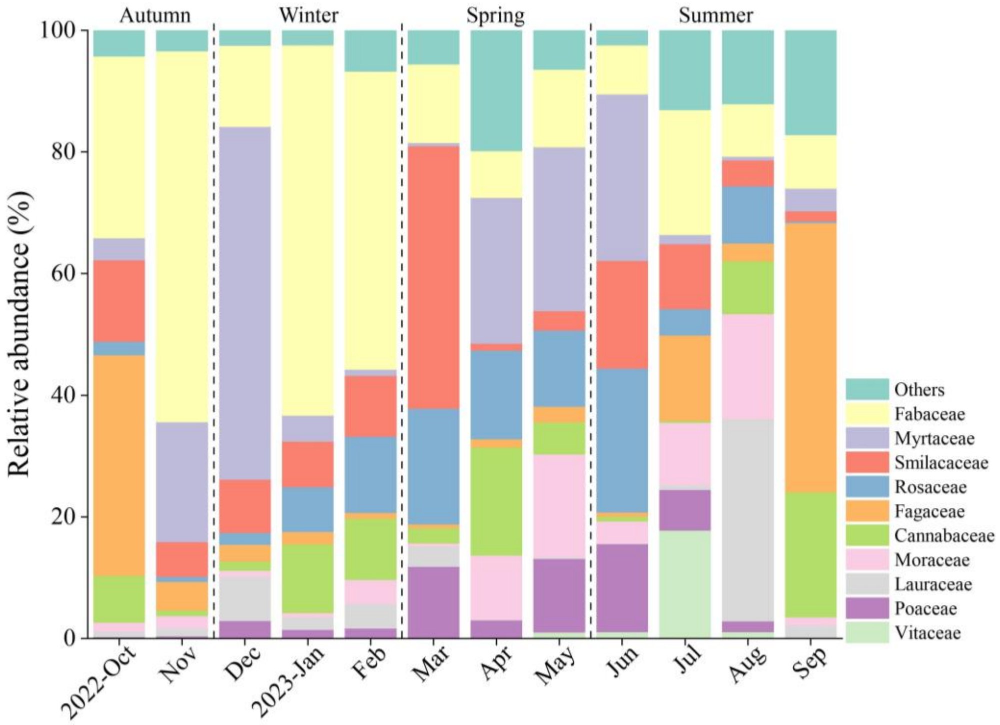

Supplementary Fig. 4 Bray-Curtis dissimilarities within seasons in dietary (A) diversity and gut microbial (B) diversity across seasons. All boxplot distributions are tested by non-parametric Kruskal-Wallis and Wilcoxon with FDR (false discovery rate) corrected *p*-value; center values indicate the median and error bars. *P<0.05, **P<0.01, ***P<0.001.

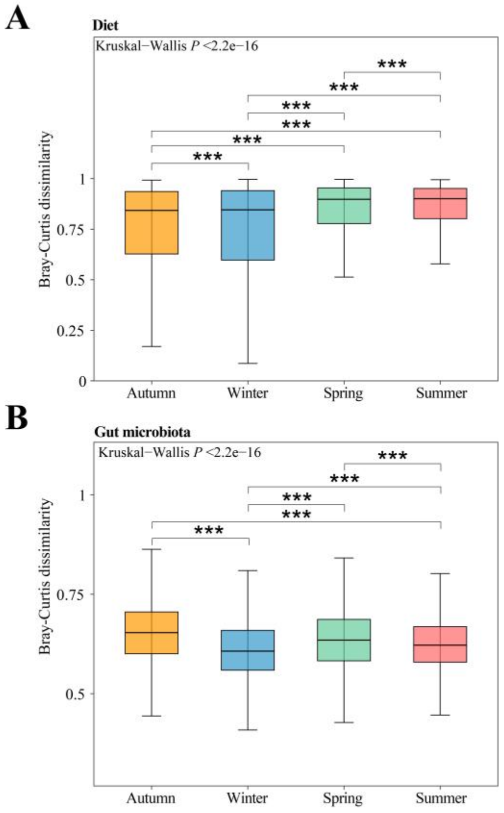

Supplementary Fig. 5 Indicator plant families and bacterial phyla that are related to each season are tracked using Sankey plots and lollipop charts. Lines represent associations between indicator plant families (**A**) or bacterial phyla (**C**) and seasons. Line width is scaled to reflect indicator value (higher indicator value indicates that the taxa was more strongly associated with that season). Lollipop charts show indicator plant families (**B**) and bacterial phyla (**D**) for the Tibetan macaque diet and gut mictobiota, respectively. A high value indicated that the taxa have a high indicator power for the corresponding season. *P*-values, * < 0.05, ** < 0.01, *** < 0.001.

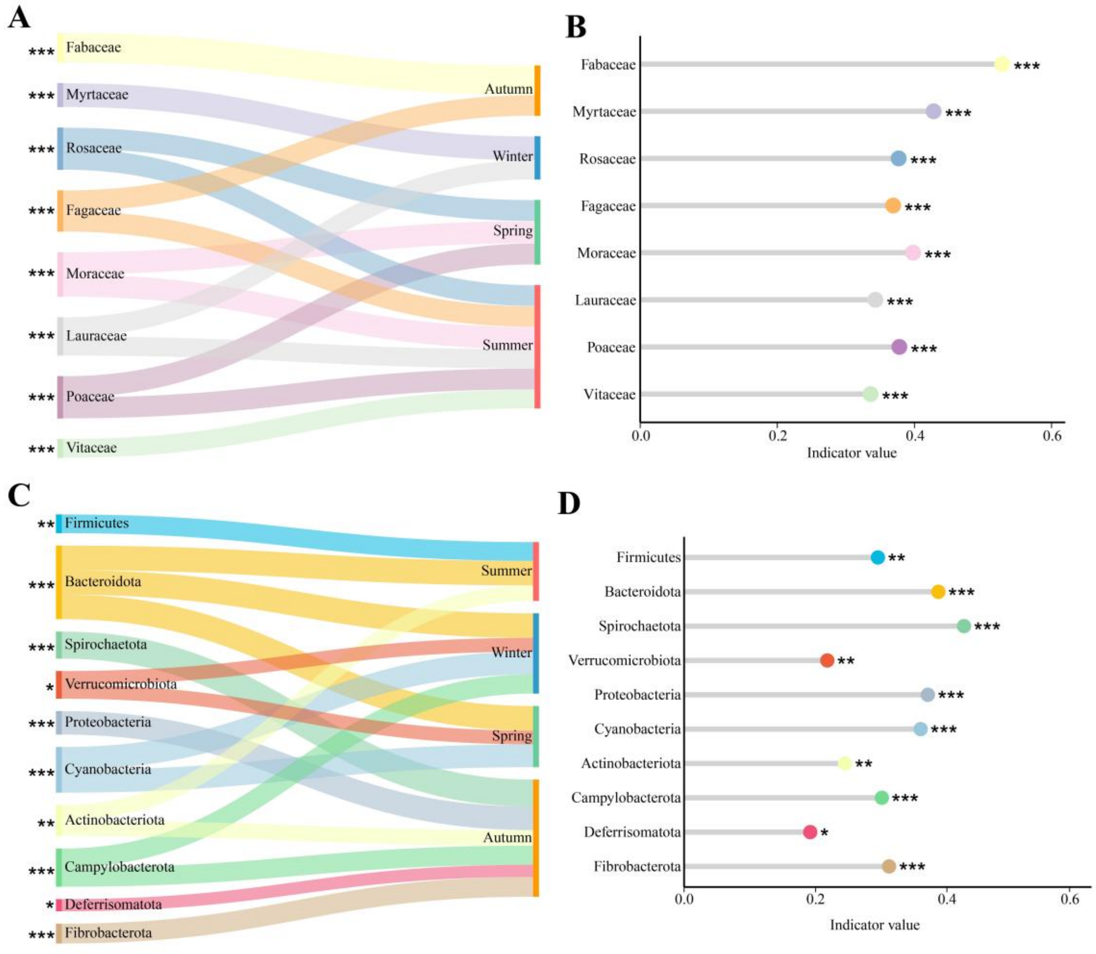

Supplementary Fig. 6 Correlation analysis among bacterial phylum and family, food types consumed, and weather conditions. The values are showen in Supplementary Table 4.

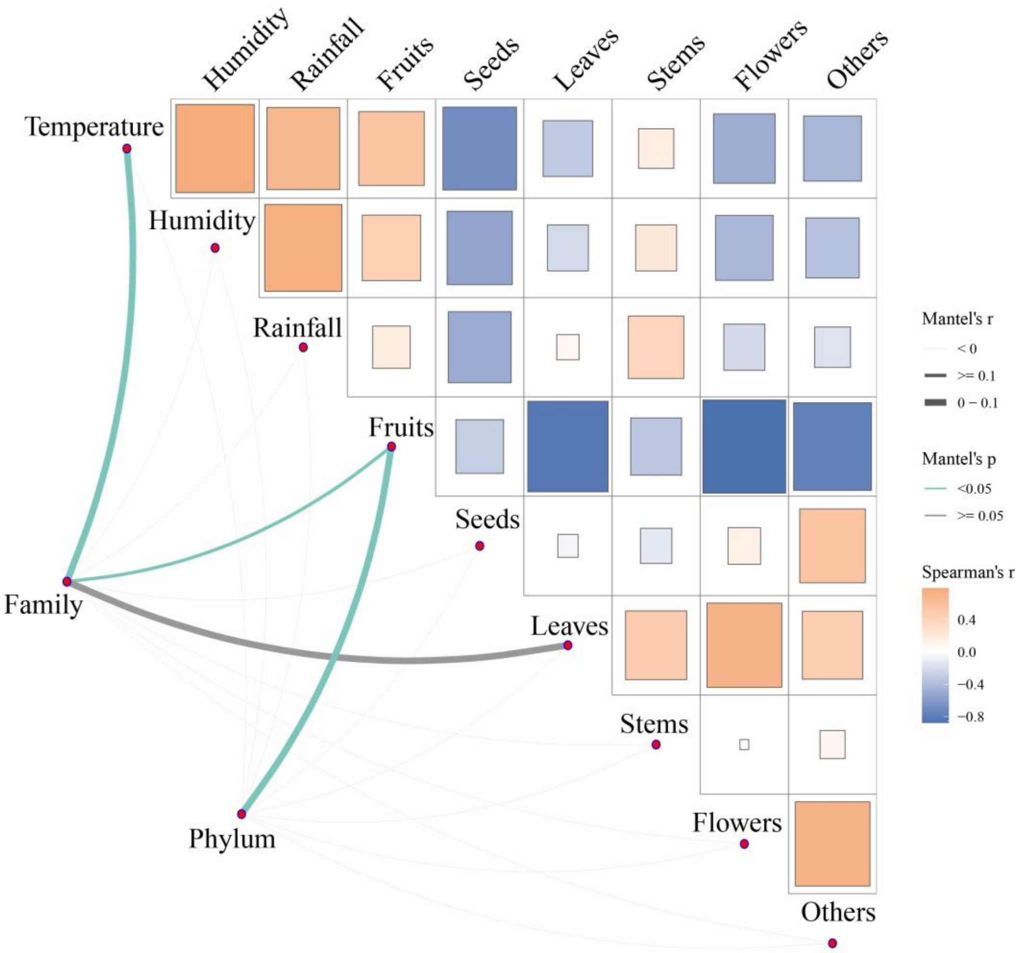

Supplementary Fig. 7 Within-season correlations between diet and gut microbiota Chao1 index, richness, and Shannon index across seasons. The significant correlation ($P < 0.05$) was bold in labels. Shaded areas represent 95% confidence intervals.

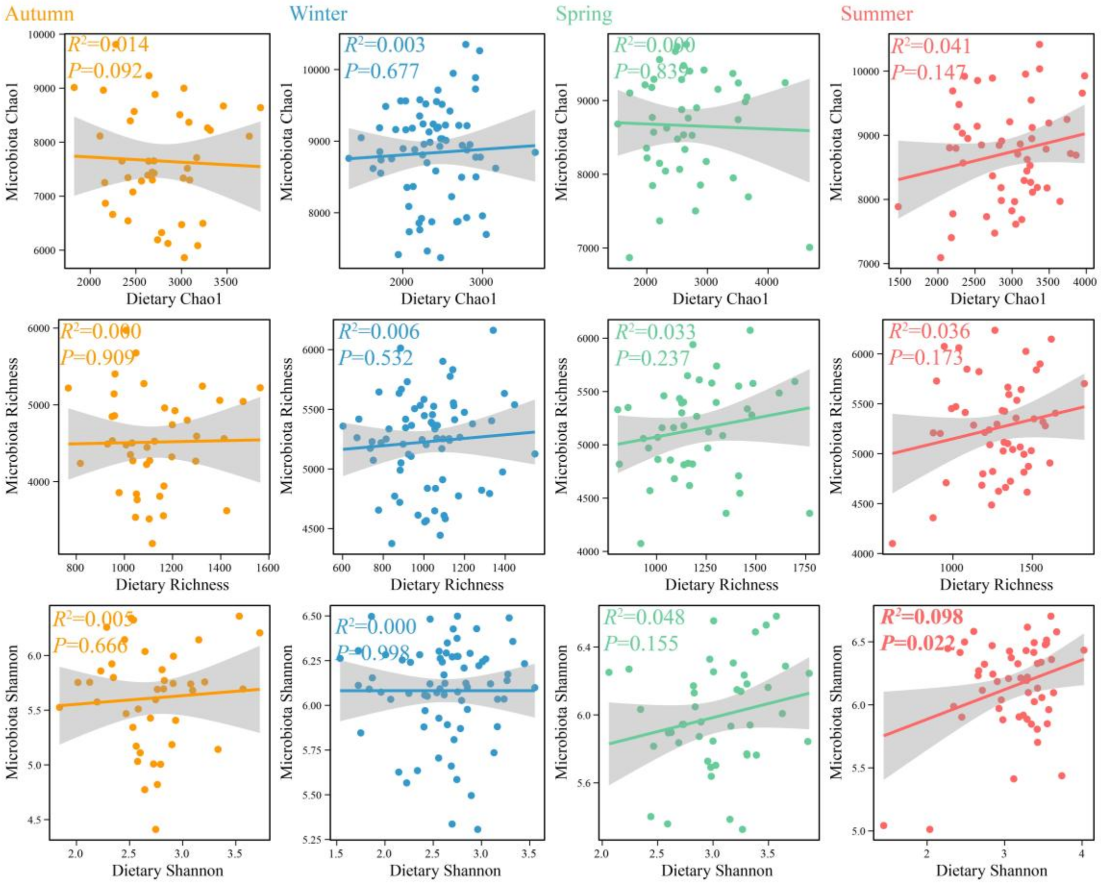

Supplementary Fig. 8 Optimal number of clusters for PAM analysis based on Jensen-Shannon distance among the relative abundance distributions at the genus level.

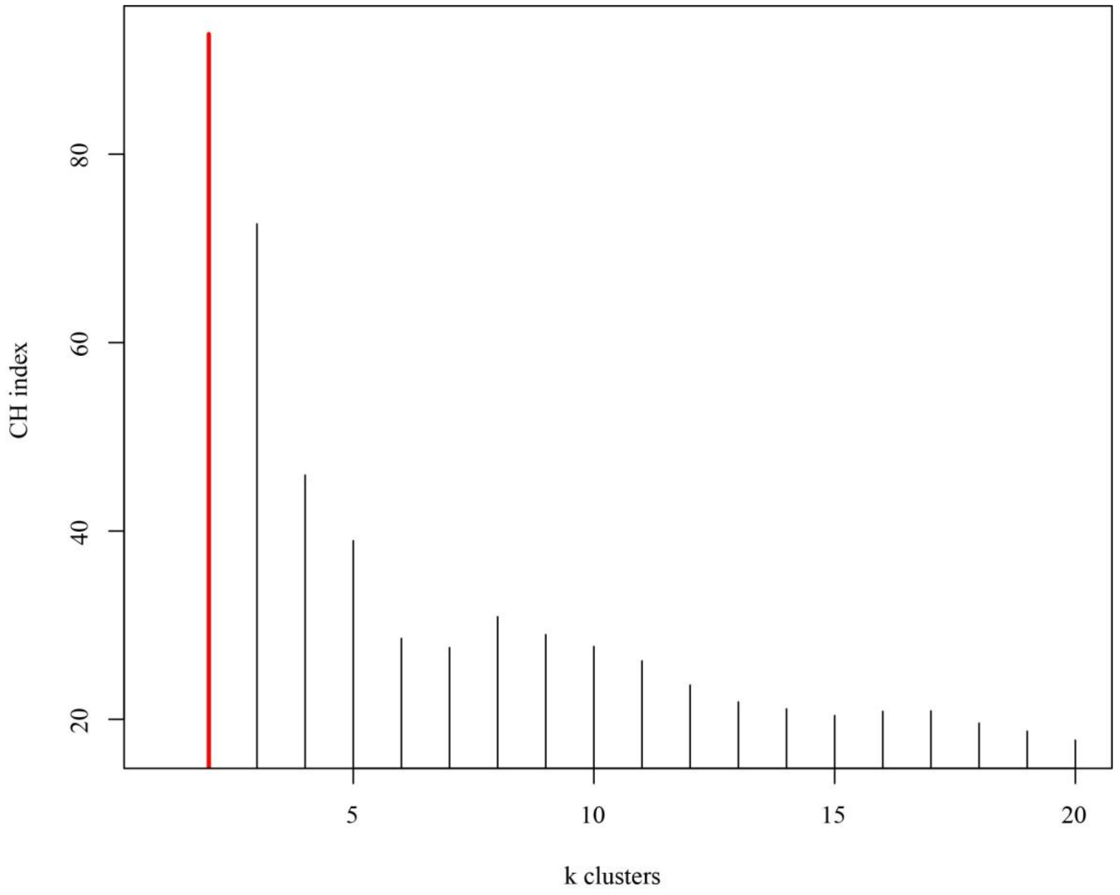

Supplementary Fig. 9 The variation in beta diversity of the gut microbiota between two enterotypes based on Bray-Curtis dissimilarity. The ellipses represent a 95% confidence interval for each group. Significance was set at the 0.05 level. The left and the upper boxplots are drawn by enterotype based on the MDS1 and MDS2 values, respectively.

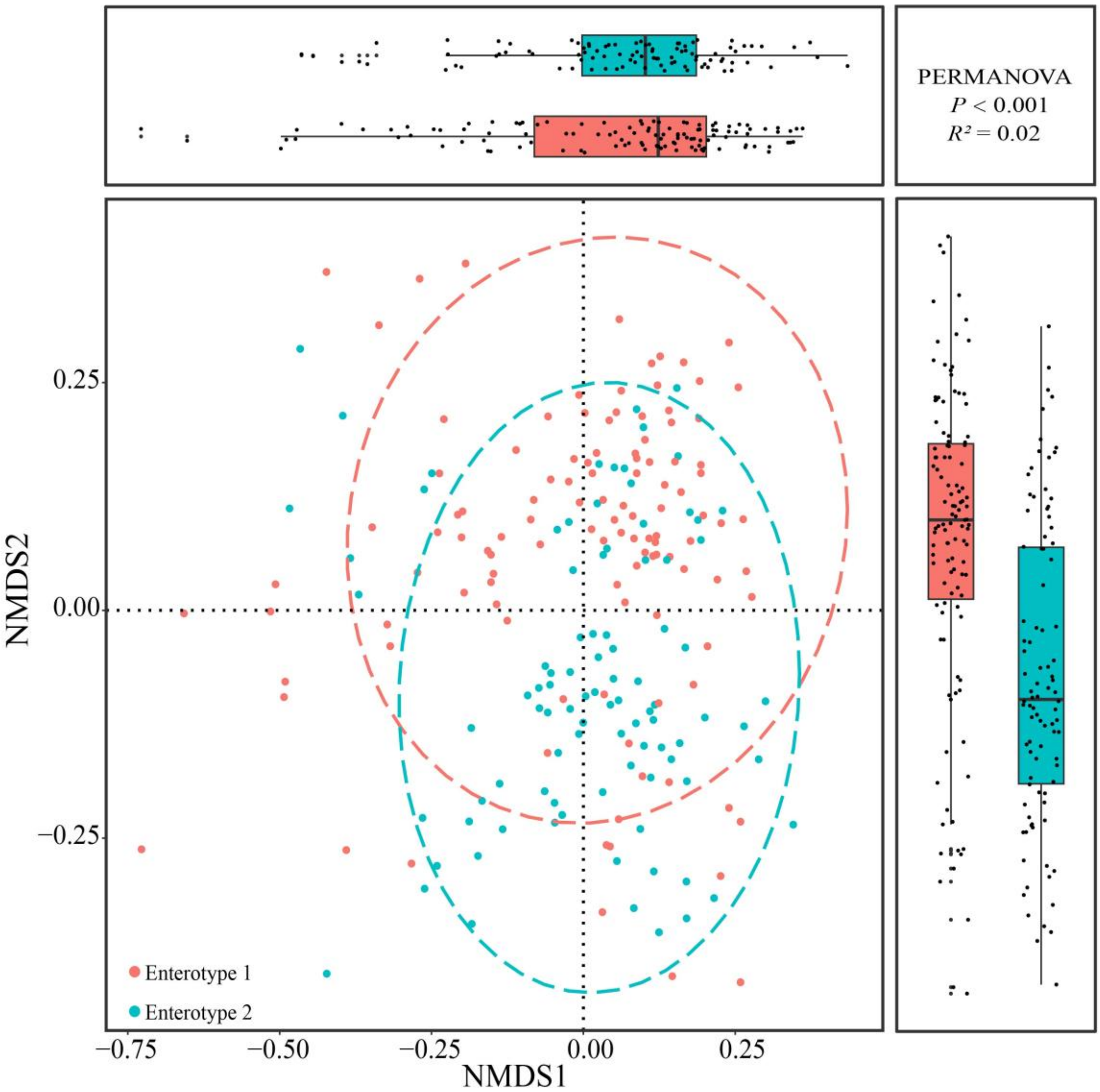

Supplementary Fig. 10 The variation in alpha diversities, including Chao1 index, richness, and Shannon index, of gut microbiota between two enterotypes. The boxplot distributions are tested using the non-parametric Kruskal-Wallis test and Wilcoxon test with FDR (false discovery rate) with corrected *p*-values. Center values indicate the median and error bars. *$p < 0.05$, **$p < 0.01$, ***$p < 0.001$.

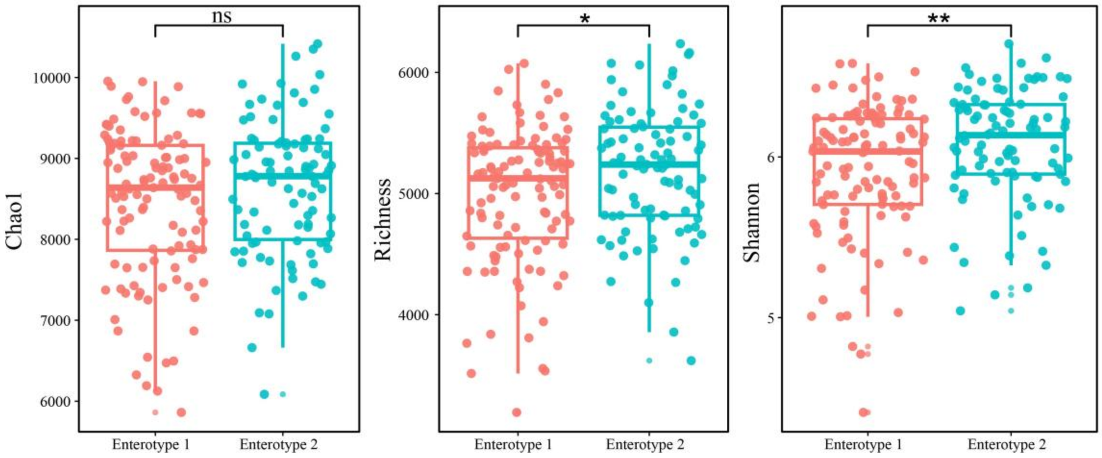

Supplementary Fig. 11 Lollipop charts show indicator bacterial genera for the gut microbiota of Tibetan macaque, with columns representing the indicator bacterial genera Lollipops are colored by different bacterial genus, and a high value indicated that the genus has a high indicator power for the corresponding group. *P*-values, * < 0.05, ** < 0.01, *** < 0.001.

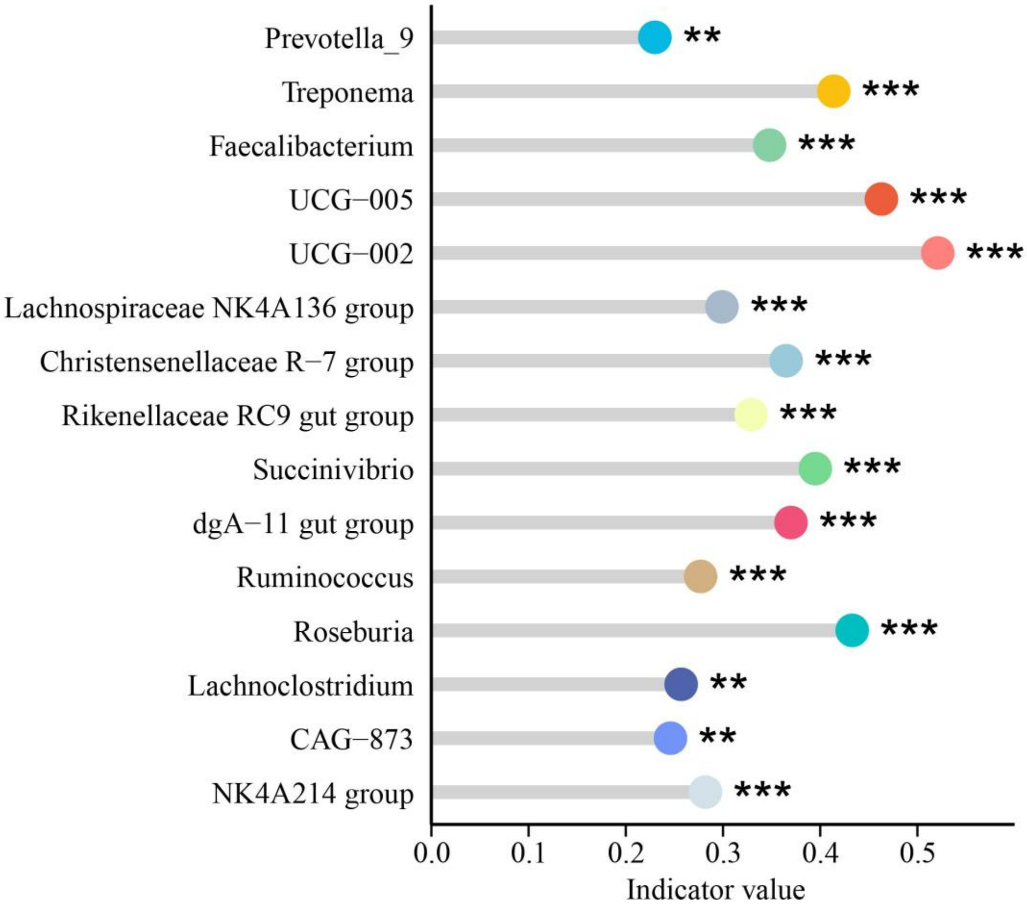

Supplementary tables

Supplementary Table 1. Monthly observation time, behavioral records, and the numbers of feces collected on Tibetan macaques at Mt. Huangshan from October 2022 to September 2023.

	Observation days	Numbers of scan	Feeding records	Numbers of feces
2022-Oct.	8	332	932	16
Nov.	9	301	678	25
Dec.	6	255	580	45
2023-Jan.	6	254	904	12
Feb.	5	183	293	14
Mar.	5	297	590	13
Apr.	4	192	661	13
May	4	212	555	18
Jun.	5	265	1010	14
Jul.	3	149	401	15
Aug.	4	175	1001	15
Sep.	3	190	1075	9
Total	62	2805	8680	209

Supplementary Table 2. The feeding time spent on top three plant items (species and types) consumed by Tibetan macaques across seasons

Season	Plant species	Food types	Percentage of records
Autumn	*Stauntonia brachyanthera*	Fruit	68.8
	Toxicodendron succedaneum	Fruit	7.3
	Castanea mollissima	Fruit	4.9
Winter	*Pinus massoniana*	Seed	36.1
	Castanopsis eyrei	Fruit	11.2
	Syzygium buxifolium	Fruit	9.1
Spring	*Cerasus serrulata*	Fruit	18.3
	Eurya nitida	Leaf	14.8
	Dalbergia hupeana	Leaf	9.0
Summer	*Castanea mollissima*	Fruit	14.9
	Lindera glauca	Fruit	14.1
	Symplocos sumuntia	Fruit	11.1

Supplementary Table 3. Seasonal abundance composition and variations of the phyla in the Tibetan macaque gut microbiota

Phylum	Autumn	Winter	Spring	Summer	*p*-value	Corrected *p*-value（FDR）
Firmicutes	48.51 ± 10.06	52.96 ± 7.09	51.68 ± 9.15	57.59 ± 9.7	< 0.001	0.001
Bacteroidota	17.95 ± 7.11	26.18 ± 7.77	25.94 ± 8.83	25.06 ± 8.31	< 0.001	< 0.001
Spirochaetota	16.42 ± 11.74	7.35 ± 6.25	9.26 ± 8.84	4.64 ± 5.49	< 0.001	< 0.001
Verrucomicrobiota	3.46 ± 2.33	5.17 ± 2.42	4.51 ± 2.16	4.11 ± 2.39	< 0.001	0.001
Proteobacteria	6.78 ± 6.78	3.01 ± 2.26	3.06 ± 2.47	3.1 ± 2.8	0.002	0.004
Cyanobacteria	0.69 ± 0.58	2.53 ± 1.89	2.14 ± 1.53	1.55 ± 1.77	< 0.001	< 0.001
Actinobacteriota	3.45 ± 6.27	0.55 ± 0.33	1.63 ± 1.4	2.23 ± 2.32	< 0.001	< 0.001
Campylobacterota	1.2 ± 1.4	1.2 ± 0.89	0.68 ± 0.62	0.55 ± 0.49	< 0.001	< 0.001
Desulfobacterota	1.4 ± 1.85	0.48 ± 0.66	0.76 ± 0.79	0.97 ± 1.05	< 0.001	0.001
Fibrobacterota	0.07 ± 0.1	0.42 ± 0.51	0.27 ± 0.27	0.2 ± 0.24	< 0.001	< 0.001
Elusimicrobiota	0.01 ± 0.04	0.15 ± 0.41	0.05 ± 0.17	< 0.01	< 0.001	< 0.001
Acidobacteriota	< 0.01	< 0.01	< 0.01	< 0.01	0.001	0.003
Chloroflexi	0.02 ± 0.05	< 0.01	< 0.01	< 0.01	< 0.001	< 0.001
Fusobacteriota	0.01 ± 0.04	< 0.01	< 0.01	< 0.01	0.001	0.002
Myxococcota	< 0.01	< 0.01	< 0.01	< 0.01	0.380	0.392
Patescibacteria	< 0.01	< 0.01	< 0.01	< 0.01	0.045	0.071
Planctomycetota	< 0.01	< 0.01	< 0.01	< 0.01	0.005	0.011
Deinococcota	< 0.01	< 0.01	< 0.01	< 0.01	0.034	0.065
Gemmatimonadota	< 0.01	< 0.01	< 0.01	< 0.01	< 0.001	0.001
Methylomirabilota	< 0.01	< 0.01	< 0.01	< 0.01	0.073	0.111
WPS.2	< 0.01	< 0.01	< 0.01	< 0.01	0.208	0.275
Bdellovibrionota	< 0.01	< 0.01	< 0.01	< 0.01	0.225	0.275
RCP2-54	< 0.01	< 0.01	< 0.01	< 0.01	0.939	0.939
Armatimonadota	< 0.01	< 0.01	< 0.01	< 0.01	0.274	0.291

Supplementary Table 3

Phylum	Autumn	Winter	Spring	Summer	p-value	Corrected p-value（FDR）
Synergistota	< 0.01	< 0.01	< 0.01	< 0.01	0.251	0.275
SAR324 clade(Marine group B)	< 0.01	< 0.01	< 0.01	< 0.01	0.041	0.069
Halanaerobiaeota	< 0.01	< 0.01	< 0.01	< 0.01	0.041	0.069
Sumerlaeota	< 0.01	< 0.01	< 0.01	< 0.01	0.006	0.013
Dependentiae	< 0.01	< 0.01	< 0.01	< 0.01	0.251	0.275
Hydrogenedentes	< 0.01	< 0.01	< 0.01	< 0.01	0.251	0.275
WS1	< 0.01	< 0.01	< 0.01	< 0.01	0.251	0.275
Latescibacterota	< 0.01	< 0.01	< 0.01	< 0.01	0.251	0.275
NB1-j	< 0.01	< 0.01	< 0.01	< 0.01	0.251	0.275
Nitrospirota	< 0.01	< 0.01	< 0.01	< 0.01	0.251	0.275
Deferrisomatota	< 0.01	< 0.01	< 0.01	< 0.01	0.041	0.069

Supplementary Table 4. Spearman correlation between environmental factors and bacterial taxanomy, including phylun, family, and genus. The values of R represent the correlation coefficients (-1 < R < 1). Negative values represent negative correlations, positive values represent positive correlations, and the greater the absolute value, the greater the correlation. ** Correlation is significant at $0.001 \leq P \leq 0.01$; * Correlation is significant at $0.01 \leq P \leq 0.05$.

Taxanomy	Environmental factors	R	P
Phylum	Temperature	-0.024	0.860
	Humidity	-0.076	0.992
	Rainfall	-0.041	0.801
	**Fruits****	**0.094**	**0.001**
	Seeds	-0.078	0.991
	Leaves	-0.036	0.823
	Stems	-0.065	0.964
	Flowers	-0.050	0.857
	Others	-0.055	0.906
Family	**Temperature***	**0.056**	**0.011**
	Humidity	-0.041	0.890
	Rainfall	-0.027	0.715
	**Fruits****	**0.143**	**0.001**
	Seeds	-0.051	0.916
	Leaves	0.051	0.098
	Stems	-0.028	0.759
	Flowers	-0.104	0.993
	Others	-0.103	0.996
Genus	**Temperature***	**0.042**	**0.038**
	Humidity	-0.034	0.838
	Rainfall	-0.029	0.721
	**Fruits****	**0.136**	**0.001**
	Seeds	-0.032	0.781
	Leaves	0.041	0.140
	Stems	-0.001	0.489
	Flowers	-0.059	0.893
	Others	-0.052	0.875

Supplementary Table 5. Spearman correlation between environmental factors and bacterial alpha diversity, including Chao1 index, richness, and Shannon diversity index.

Bacterial alpha diversity	Environmental factors	R	P
Chao1 index	Temperature	-0.056	0.418
	Humidity	-0.068	0.326
	Rainfall	0.060	0.388
	**Fruits****	**-0.204**	**0.003**
	**Leaves****	**0.206**	**0.003**
	Stems	0.107	0.124
	**Flowers***	**0.152**	**0.028**
	Others	0.119	0.086
Richness	Temperature	0.026	0.705
	Humidity	0.004	0.949
	Rainfall	0.081	0.246
	**Fruits***	**-0.177**	**0.010**
	**Leaves****	**0.195**	**0.005**
	Stems	0.103	0.138
	Flowers	0.095	0.170
	Others	0.074	0.287
Shannon diversity index	Temperature	0.074	0.286
	Humidity	0.014	0.836
	**Rainfall***	**0.175**	**0.011**
	**Fruits***	**-0.144**	**0.038**
	**Leaves***	**0.158**	**0.022**
	Stems	0.106	0.126
	Flowers	0.052	0.456
	Others	0.060	0.386
	Temperature	-0.056	0.418
	Humidity	-0.068	0.326
	Rainfall	0.060	0.388

Supplementary Table 6. Comparison of the dissimilarity of the Tibetan macaque gut microbiota or plant-based diets within a season and between seasons

Within season	Between season	W-statistic	*P*-value	Adj *P*-value	Note
Within autumn	Between autumn and winter	857310.5	< 0.001	< 0.001	Bacterial Bray-Curtis dissimilarity
Within autumn	Between autumn and spring	454760.5	< 0.001	< 0.001	Bacterial Bray-Curtis dissimilarity
Within autumn	Between autumn and summer	583765.5	< 0.001	< 0.001	Bacterial Bray-Curtis dissimilarity
Within winter	Between autumn and winter	1000445.5	< 0.001	< 0.001	Bacterial Bray-Curtis dissimilarity
Within winter	Between winter and spring	1622598	< 0.001	< 0.001	Bacterial Bray-Curtis dissimilarity
Within winter	Between winter and summer	1712970.5	< 0.001	< 0.001	Bacterial Bray-Curtis dissimilarity
Within spring	Between autumn and spring	490555	< 0.001	< 0.001	Bacterial Bray-Curtis dissimilarity
Within spring	Between winter and spring	1270927	< 0.001	< 0.001	Bacterial Bray-Curtis dissimilarity
Within spring	Between winter and summer	989684.5	< 0.001	< 0.001	Bacterial Bray-Curtis dissimilarity
Within summer	Between autumn and summer	639803	< 0.001	< 0.001	Bacterial Bray-Curtis dissimilarity
Within summer	Between winter and summer	1680783	< 0.001	< 0.001	Bacterial Bray-Curtis dissimilarity
Within summer	Between winter and summer	1071810	< 0.001	< 0.001	Bacterial Bray-Curtis dissimilarity
Within autumn	Between autumn and winter	848801.5	< 0.001	< 0.001	Plant-based Bray-Curtis dissimilarity
Within autumn	Between autumn and spring	119125.5	< 0.001	< 0.001	Plant-based Bray-Curtis dissimilarity
Within autumn	Between autumn and summer	134533	< 0.001	< 0.001	Plant-based Bray-Curtis dissimilarity
Within winter	Between autumn and winter	2907310	< 0.001	< 0.001	Plant-based Bray-Curtis dissimilarity
Within winter	Between winter and spring	921161	< 0.001	< 0.001	Plant-based Bray-Curtis dissimilarity
Within winter	Between winter and summer	980247.5	< 0.001	< 0.001	Plant-based Bray-Curtis dissimilarity
Within spring	Between autumn and spring	388028.5	< 0.001	< 0.001	Plant-based Bray-Curtis dissimilarity
Within spring	Between winter and spring	672094.5	< 0.001	< 0.001	Plant-based Bray-Curtis dissimilarity
Within spring	Between winter and summer	911832	< 0.001	< 0.001	Plant-based Bray-Curtis dissimilarity
Within summer	Between autumn and summer	795582	< 0.001	< 0.001	Plant-based Bray-Curtis dissimilarity
Within summer	Between winter and summer	1316898.5	< 0.001	< 0.001	Plant-based Bray-Curtis dissimilarity
Within summer	Between spring and summer	1560223.5	0.140	0.140	Plant-based Bray-Curtis dissimilarity

Supplementary Table 7. Comparison of the dissimilarity between gut microbiota and plant-based diet

Within/between seasons	Mean weighted Bray-Curtis dissimilarities of gut microbiota	Mean weighted Bray-Curtis dissimilarities of plant-based diets	W-statistic	*P*-value	Adj *P*-value
Within autumn	0.610	0.570	366141	0.002	0.002
Within winter	0.552	0.600	2801164	< 0.001	< 0.001
Within spring	0.611	0.747	224078	< 0.001	< 0.001
Within summer	0.587	0.789	280809	< 0.001	< 0.001
Between autumn and winter	0.655	0.682	3613828	< 0.001	< 0.001
Between autumn and spring	0.671	0.896	202742	< 0.001	< 0.001
Between autumn and summer	0.663	0.899	169989	< 0.001	< 0.001
Between winter and spring	0.631	0.895	548888	< 0.001	< 0.001
Between winter and summer	0.626	0.907	141643	< 0.001	< 0.001
Between spring and summer	0.627	0.811	653838	< 0.001	< 0.001
