# Supplementary material for: Stability and flexibility of the gut microbiota of wild Tibetan macaques
Source: ISME Commun. 2025 Nov 26;5(1):ycaf184. doi: 10.1093/ismeco/ycaf184 (PMC12648401; doi:10.1093/ismeco/ycaf184)
Supplement: Supplementary_Methods_ycaf184 [file supplementary_methods_ycaf184.pdf]

Supplementary methods

Constructing the local reference database

To maximize identification accuracy and minimize interference from non-local related species (or genera) during sequence alignment, this study first established a local plant DNA barcode reference database. Subsequently, based on the book Plants of Huangshan Mountain, a comprehensive list of local plant species was compiled. We then queried the NCBI database for records of plant-matching "*trn*L" sequences at the species level. Finally, the *trn*L sequence information of these plants was retrieved and downloaded, saved in a FASTA format file, and the barcode reference database was constructed using the local BLAST+ tool.

Taxon Annotation Principles

The *trn*L barcode sequencing results were compared for similarity against the established plant databases, with the principles for taxon annotation being: (1) If the similarity is ⩾ 98% to a single species sequence, it was recorded as that species. (2) If the similarity is ⩾ 98% to sequences of multiple species, it was recorded as the lowest taxonomic unit that covers all species with ⩾ 98% similarity. (3) If the highest similarity is < 98% but ⩾ 95%, and there is only one sequence with the highest similarity and the difference in similarity to the next highest scoring sequence is ⩾ 1%, it was recorded as the higher taxonomic unit of the sequence with the highest similarity. If the highest similarity corresponds to multiple species, it was recorded as the lowest taxonomic unit that covers all species with the highest similarity. (4) If the highest similarity was < 95%, it was considered that the taxonomic unit of this sequence could not be determined and was not included in the dietary results.
